# F226. CLINICAL FACTORS ASSOCIATED WITH CONTINUATION OF LONG-ACTING INJECTABLE ANTIPSYCHOTIC MEDICATION: RETROSPECTIVE CHART REVIEW

**DOI:** 10.1093/schbul/sby017.757

**Published:** 2018-04-01

**Authors:** Shi Hyun Kang, Jong Il Lee

**Affiliations:** Seoul National Hospital

## Abstract

**Background:**

Long-acting injectable (LAI) antipsychotic medications provide a potential solution to the poor adherence to oral therapies in schizophrenia. However, not all patients with schizophrenia seem to have therapeutic benefits using LAI antipsychotics.

The objectives of this study were to investigate clinical factors in patients with schizophrenia who have continued the LAI antipsychotics compared with the patients who have discontinued the LAI antipsychotics.

**Methods:**

Data were collected by retrospective chart review of all 150 patients prescribing LAI therapy during 2005–2012 in a mental hospital. The data including age at onset, age at starting LAI antipsychotics, duration of illness at starting LAI antipsychotics, total number of admission before starting LAI antipsychotics were gathered. The subjects were classified into three group; 1) the continuation group (n=27, 18.0%), 2) the discontinuation group (n=57, 38.0%), and 3) the follow-up loss group (n=66, 44%). The ANOVA were used to compare the clinical variables among 3 groups. The stepwise multiple linear regression analyses were conducted to evaluate the association between the duration of LAI medications and clinical variables.

**Results:**

There were significant differences among three groups in age (52.9 ± 9.1, 44.8 ± 12.9, 49.5 ± 12.0, p=0.009), age at onset (30.7 ± 10.4, 24.6 ± 9.9, 26.2 ± 8.6, p=0.027) and age at starting LAI medications (44.1 ± 8.9, 37.0 ± 12.4, 40.8 ± 11.2, p=0.021). In regression analyses, the duration of LAI medications were significantly associated with age (β=1.727, p<0.001), age at starting LAI medications (β=-1.489, p<0.001), and the total number of admission before starting LAI medications (β=-0.177, p=0.008).

**Discussion:**

The continuation of LAI medications was associated with age, age at starting LAI medications and the number of admission before starting LAI medications.

